# Foamy Melamine Resin–Silica Aerogel Composite-Derived Thermal Insulation Coating

**DOI:** 10.3390/nano15020135

**Published:** 2025-01-17

**Authors:** Dongfang Wang, Yabin Ma, Yingjie Ma, Baolei Liu, Dewen Sun, Qianping Ran

**Affiliations:** 1 State Key Laboratory of High-Performance Civil Engineering Materials, Jiangsu Sobute New Materials Co., Ltd., Nanjing 210008, China; mayabin@cnjsjk.cn (Y.M.); mayingjie@cnjsjk.cn (Y.M.); liubaolei@cnjsjk.cn (B.L.); sundewen@cnjsjk.cn (D.S.); ranqianping@cnjsjk.cn (Q.R.); 2 School of Materials Science and Engineering, Southeast University, Nanjing 211189, China

**Keywords:** thermal insulation, aerogel composite, coating

## Abstract

A novel class of SiO_2_ aerogel-based resin composite with a self-formed foamy structure and an extremely low thermal conductivity, as well as excellent fire resistance, was fabricated via a room temperature and atmospheric pressure route. The self-formed foamy structure was achieved by utilizing SiO_2_ aerogel particles not only as a thermal insulative functional additive filler but also as nano-sized solid particles in a Picking emulsion system, adjusting the surface tension as a stabilizer at the interface between the two immiscible phases (liquid and air in this case). The results of foamy structure analyses via scanning electron microscopy, micro-CT, and N_2_ adsorption–desorption isotherms validate the successful generation of a micro-scale porous structure with the enhancement of the aerogel nano-scale solid particles at the wall as a stabilizer. A combination of multiscale pores imbues the aerogel-based foamy coating with a low thermal conductivity, as well as a high cohesive strength. For the foamy coating studied, with variable emulsion/foaming agent/aerogel ratios of 1/2/x, the thermal conductivity decreases from 0.141 to 0.031 W/m·K, and the cohesive strength increases from being non-detectable to 0.41 MPa. The temperature difference, which is a direct indicator of the thermal insulation behavior of the foamy coating, can increase from 12.1 °C to 48.6 °C under an 80 °C hot plate.

## 1. Introduction

Efficient thermal energy management is critical in advancing technologies like thermoelectric generators and in efforts to achieve carbon neutrality [1,2,3]. In the building sector, as the demand for energy-efficient buildings grows, particularly in renovating older structures, the need for high-performance exterior thermal insulation coatings has become more pressing. Traditional thermal insulation materials, such as pads, often have significant drawbacks, including flammability, high carbon emissions, and limited structural integrity. In contrast, thermal insulation coatings [4,5,6] based on polymer primers and aerogels may offer outstanding thermal insulation properties, excellent environmental durability, and a high cohesive strength.

Recent developments in materials like melamine–formaldehyde (MF) resin and silica aerogel offer promising alternatives. Melamine–formaldehyde (MF) resin is a resin characterized by melamine rings terminated with multiple hydroxyl groups, which are derived from formaldehyde [7]. One of the primary applications of MF resin is its use as a coating agent for surface protection. Recent studies have also confirmed the resin’s potential in carbon dioxide capture. The density of MF resin (30–115 kg/m^3^) is relatively low compared to that of other major resins. Additionally, MF resins exhibit low thermal conductivity, excellent fire resistance, and a low carbon emission rate [8,9], making them a promising candidate for use in external building thermal insulation materials. Silica aerogel was first synthesized in 1931 [10] and is renowned for its ultra-low density, three-dimensional nanoporous structure, high specific surface area, and low thermal conductivity [11,12,13,14]. Additionally, SiO_2_ aerogels are highly stable, non-combustible, and resistant to most chemicals, making them suitable for various demanding insulation applications. These properties arise from their unique nanoporous structure, which significantly reduces heat transfer through conduction, convection, and radiation [15,16] and also supports their applications in environmental cleanup [17,18,19] and drug delivery [20,21]. Together, these materials offer great potential for creating advanced thermal insulation coatings with a superior performance.

However, due to the unique nanostructure and surface properties of silica aerogel powder, achieving a uniform dispersion of silica aerogel powder in waterborne emulsions is challenging, thus limiting the development of aerogel-based coatings. Furthermore, directly mixing SiO_2_ aerogel particles with polymer emulsions can cause intrusion of the primer into the aerogel’s porous structure. Researchers have analyzed the interaction between aerogel particles and epoxy resin in a mechanical context. Resin with a relatively low viscosity can cause significant intrusion into the aerogel’s porous structure, leading to structural and functional failure when it is mixed with aerogel particles [22]. This intrusion may lead to the collapse of the open porous structure, reducing the porosity, increasing the density, and impairing the thermal insulating properties. According to research by Saeed [23], the intrusion of the emulsion into the pores of the aerogel leads to no reduction in thermal conductivity, even though a significant enhancement in the mechanical properties is achieved. Therefore, careful design of the aerogel-based composite’s microstructure is crucial for developing effective aerogel-based thermal insulation coatings in the coating industry.

The stabilizing capability of solid particles in foams and emulsions was first realized in 1904 as Pickering emulsions [24,25,26,27,28]. Similarly, a Pickering foam is a dispersed system where gas bubbles in a liquid are stabilized by solid particles [29,30,31,32,33,34]. A wide range of inorganic particles, such as silica [33,35], titanium dioxide [36], and calcium carbonate [37], have been approved for use in stabilizing Pickering foams [38]. Research has demonstrated that partially hydrophobic nanoparticles have significant potential as stabilizing agents in various foaming processes [39]. These nanoparticles exhibit behavior similar to that of polymer surfactants, as they attach to the bubble–liquid interface and provide long-term stability for extended periods, sometimes lasting up to several years. The unique properties of nanoparticles make them highly effective at reducing surface tension and preventing bubble coalescence. This is crucial in a variety of industrial applications, including food processing, energy-efficient building materials, and thermal management systems.

Based on the above background, our work utilizes partially hydrophobic silica aerogel powder as an interface stabilizer during the formation process for MF foam. This approach ensures even mixing of the two components and results in a hierarchical micro/nanoporous structure in the final product.

## 2. Materials and Methods

### 2.1. Materials

Melamine–formaldehyde resin (MF resin) was obtained from Shandong Jiaying Chem. Co., Ltd. (Zibo, China) without further purification needed before its use. *p*-Toluenesulfonic acid (TsOH, 99%), sodium alginate (SA), and whey protein (80%) were purchased from Shanghai Macklin Biochemical Co. Ltd. (Shanghai, China) Gum Arabic (GA, 99%) was obtained from Nexira Inc., Somerville, NJ, USA. Hydrophobic silica aerogel with a particle diameter of around 15 μm was obtained from Suzhou Tenanom Tech Co. Ltd., Suzhou, China, and was used as is without further treatment.

### 2.2. Methods

#### 2.2.1. Preparation of the Melamine Resin Aerogel

A 4 wt.% foaming agent (SA or GA) aqueous solution was prepared and allowed to settle overnight to ensure its complete dissolution. *p*-Toluenesulfonic acid (TsOH) was dissolved in distilled water at a 50 wt.% concentration to serve as the curing agent for the MF resin. The MF resin was then mixed with the curing agent solution at a 4:1 ratio to form an MF solution. To enhance the foaming properties, 1 wt.% whey protein was added to the foaming agent solution, resulting in an enhanced foaming solution. The MF solution, the enhanced foaming solution, and the SiO_2_ aerogel powder were mixed at a specific mass ratio under 700 rpm for 20 min, yielding an MF aerogel foam. The mixture was then cured at 60 °C for 24 h to accelerate the curing process and enhance the stability of the foamy structure. After curing, the melamine resin–silica aerogel composite samples were obtained and labeled as MSA-x/y/z and MGA-x/y/z. Here, “M” represents melamine resin, followed by the type of foaming agent used (SA or GA), and x/y/z denotes the proportion of the three main components in the composite: MF resin, the 4 wt.% foaming agent solution, and silica aerogel powder, respectively. A detailed list of the samples is addressed in Table 1 below.

#### 2.2.2. Measurements and Characterization

To determine the porous structure, surface area, and density of the studied composite, nitrogen gas adsorption tests were undertaken using a Brunauer–Emmet–Teller analysis (BET; TriStar II 3020, Micromeritics, Norcross, GA, USA). The degassing of the samples was carried out under a vacuum for 2 h at 90 °C, which was lower than the degradation temperature. A N_2_ adsorption–desorption analysis was undertaken using an automatic system. The specific surface area (S_BET_) of the foamy melamine resin–silica aerogel composite was determined using the Brunauer–Emmett–Teller (BET) theory, which relies on N_2_ adsorption measurements. The pore size distribution was plotted according to the NLDFT analysis, assuming a cylindrical pore geometry to fit the adsorption–desorption isotherms. The average pore diameter (L) was calculated using the simplified formula L = 4·V_T_/S_BET_, where V_T_ is the total pore volume, and a cylindrical pore shape is assumed.

The surface morphology of the foamy melamine resin–silica aerogel composite was analyzed using scanning electron microscopy (Hitachi Regulus8100 (Tokyo, Japan)). Due to the low electrical conductivity of the samples, the sample surface was coated with a thin layer of platinum before the SEM measurements. A micro-CT analyzer (ZEISS Xradia Context micro-CT (Oberkochen, Germany)) was used to visualize the samples and calculate the parameters of the foamy aerogel coating samples with a step size of 3.6 µm, an exposure time of 0.26 s, and a transmittance of 0.8, and no filter was used.

**Figure 1:**
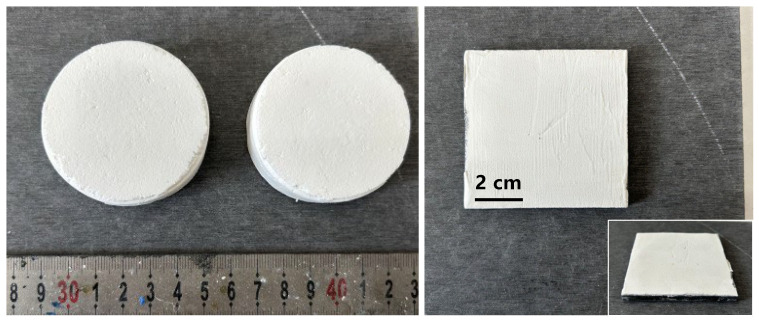
Completely dried MGA-10/20/5 in a cylinder shape (**left**) and the 1 mm foamy aerogel composite as a coating on fiber–cement board (**right**).

The thermal conductivity of the samples of the foamy aerogel composites was investigated using guarded hot plate apparatus, manufactured by Impal Measurement and Control Equipment Co., Ltd., Guangzhou, China, which complies with the GB/T 10294-2008/ISO 8302:1991 standard [40]. The thermal conductivity measurements were taken at the designated temperature using two identical specimens (300 mm × 300 mm × 15 mm) which had been thoroughly dried beforehand. Temperature difference tests were conducted on MGA series coatings of a 1 mm thickness applied to a fiber–cement board (FCB), as indicated in Figure 1 (right). An infrared thermal imager was used to visualize the thermal insulation performance of the foamy aerogel. For comparison, an FCB coated with a regular MF resin coating of the same thickness, but without the foamy structure, was also tested. A heat plate at 80 °C was applied to the bottom surface of the FCB for 10 min, and the temperature of the top surface was recorded. Whenever possible, results averaged from three replicate samples were reported as the representative cohesive strength. It was critical to ensure that the end surfaces of the samples were smooth and parallel. For this purpose, the end surfaces were polished with sandpaper. All the above analyses were carried out thrice within different regions of interest to satisfy the principle of parallel repetition.

## 3. Results and Discussion

### 3.1. Surface Area and Porosity

**Figure 2:**
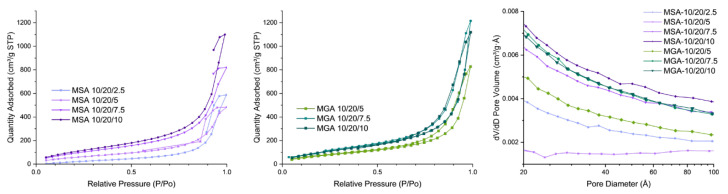
Relative pressure distribution of MSA 10/20/x series (**left**) and MGA 10/20/x series (**center**) and pore diameter distribution (**right**) of 2 foamy aerogel composites.

Figure 2 and Table 2 present the BET isotherms of the foamy aerogel coatings. As can be observed from the adsorption–desorption isotherm curves, in a P/P_0_ range from 0.65 to 1.0, the changing rate of the adsorbed quantity increases quickly and sharply, and a hysteresis circle forms between the adsorption and desorption curves, indicating the foamy coatings with a hierarchical macro-/microporous structure are starting to absorb/desorb gas from the inflection point. The samples with SA as the foaming agent are thick and form a foam structure with difficulty; thus, the surface area is much lower than that of those with GA as the foaming agent. As shown in Figure 2 (left), the isotherm of the MSA 10/20/x composite series has no closed loop, which indicates that the pore structure that was created using SA as the foaming agent cannot withstand the N_2_ adsorption–desorption pressure and non-reversible collapse occurred in this series of samples. When GA was selected as the foaming agent for the MGA 10/20/x series, all of the samples exhibited type IV isotherms with an H3 hysteresis loop [41], which indicates that they contained a large number of mesopores, and the pore size was mainly distributed in the range of 3–8 nm (Figure 2, left).

### 3.2. Microstructure

**Figure 3:**
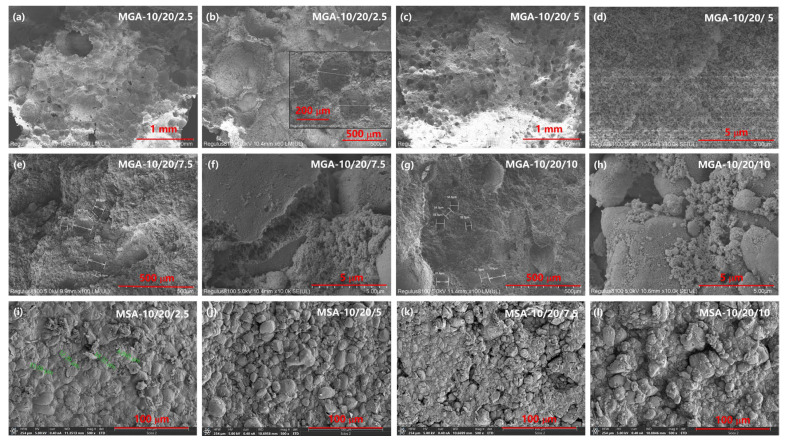
SEM images of foamy aerogel coatings with GA (**a**–**h**) and SA (**i**–**l**) as foaming agents.

After complete curing and hardening, the hierarchical porous structure is well preserved, with a mesopore size ranging from 3 to 8 nm, as indicated in the BET pore volume data, and a micropore size in the range of 1–10 μm can be concluded from the SEM images, as well as the micro-CT analyses. Figure 3 displays the scanning electronic microscopy images (SEM) of the foamy aerogel coatings based on the different foaming agents, GA and SA, respectively. Considerable changes can be observed on the basis of the variation in the foaming agent concentrations. From the images shown in Figure 3i–l, it can be observed that the segment surfaces of the aerogel composites with SA as the foaming agent are bumpy and condensed, with no obvious foamy structure observed in various MSA composites. Moreover, the size of the bumpy texture shows a decreasing trend as more aerogel particles are incorporated into the composites. Figure 3a–h show the section surfaces of the foamy aerogel coatings with an increasing amount of GA as the foaming agent. Compared to SA, GA has a stronger ability to hold water and to generate a microscale foamy structure, as well as preserve the nano-scale pore structure in the SiO_2_ aerogel; thus, the GA-based foamy coatings have an obviously hierarchical foamy structure from the SEM images of the section surfaces. As the filling amount of aerogel particles increases, the foamed pore diameter of the foamy coatings gradually decreases, and the wall thickness between pores gradually increases. From the broken gap in the resin-warped aerogel (Figure 3f), which is the main component of the walls between the pore structure, the internal structure of the aerogel particles is well preserved and relatively complete. It can be observed that not too many of the nanopores in the aerogels were filled with resin, which proves the feasibility of the protective effect of the foamed resin–aerogel composite thermal insulation coating on the overall pore structure of the aerogel.

**Figure 4:**
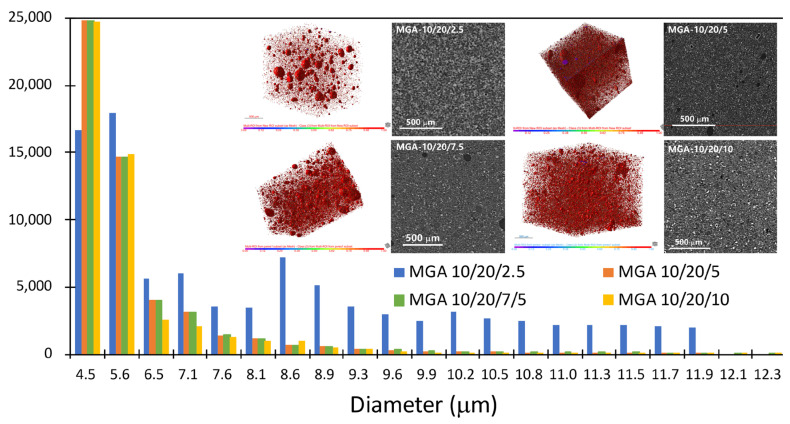
Pore size distribution of the MGA-10/20/x foamy aerogel coating from the micro-CT analysis.

Micro-CT analyses of the foamy aerogel composites that were prepared with GA are presented in Figure 4. An obvious porous structure can be observed in all samples with various resin/foaming agent/aerogel ratios. When the amount of aerogel is low, such as in MGA 10/20/2.5, the pore size distribution is wide and irregular. With an increasing amount of aerogel involved in the system, the size distribution in the porous structure becomes more and more centralized and uniform. Thus, the aerogel particles not only serve as a foaming agent but also act as a distributor and stabilizer in the composite.

The pore size distribution analysis reveals that the majority of the pore volume is concentrated in the range of 4.5–5.6 μm, with variations in the pore count and size distribution observed across different samples. Specifically, the MGA-10/20/2.5 sample exhibits the highest pore volume within this range, while an increasing ratio (e.g., MGA-10/20/7.5 and MGA-10/20/10) leads to a reduction in the pore volume and a more uniform distribution. This trend suggests that modification of the synthesis conditions influences the material’s porosity, resulting in finer and more compact structures. Corresponding microstructural images further confirm this observation, showing larger, well-defined pores in lower-ratio samples and a denser pore network in higher-ratio samples. Such a hierarchical pore structure, combining micro- and mesopores, likely enhances the material’s functional properties, such as its thermal insulation or specific surface area, making it suitable for advanced applications.

Contrary to the use of defoaming agents in the general coating preparation procedure, a foaming agent is the key component here in this research. With abundant air bubbles in the precursor slurry under vigorous mechanical stirring, the liquid–air interfaces keep foaming, and aerogel nanoparticles constantly evolve into these interfaces. When the aerogel content is low, Ostwald ripening causes the gas in the small pores to form large bubbles under the driving force of the Laplace pressure difference between bubbles [42]. As a result, large bubbles grow and tend to broaden the pore size distribution. However, as the amount of aerogel nanoparticles increases, with the solvent evaporating, the aerogel particles gradually lose their mobility and aggregate as walls among the bubbles, stabilizing the micro-scale porous structure before complete drying of the composite, as reported by previous studies [43,44,45,46].

Consequently, the pore size and the wall structure are tunable with the MF resin and aerogel content. As the amount of aerogel increases, the pore size of the composite increases, accompanied by the evolution of the wall structures from a thin necklace structure to a truss structure. The amount of aerogel contributes to the formation of a continuous aerogel layer. The aerogel–resin-based truss skeleton stands out for constructing a low-density and robust structure that is necessary and critical to protect the pores from stress-induced collapse. Thus, the thermal insulation properties and mechanical properties of the foamy aerogel can be tuned and controlled.

### 3.3. Thermal Properties

**Figure 5:**
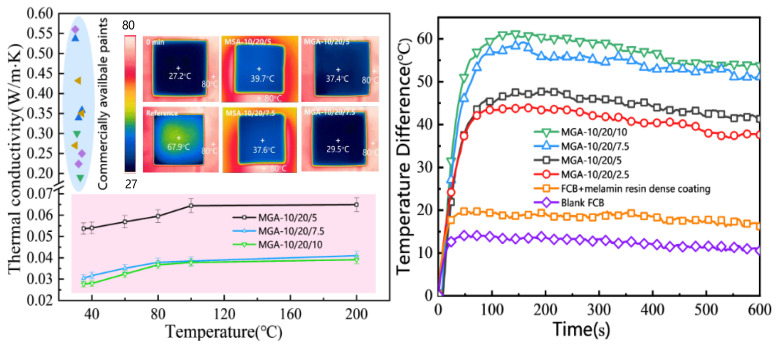
Thermal conductivity of MGA-10/20/x samples ((**left**) (points in blue shade are thermal conductivities of some commercially available paints)), thermal insulation behavior (left inset), and temperature difference in MGA-10/20/x samples (**right**).

The measured thermal conductivity of the foamy aerogel composites ranges from 30 to 110 mW/m·K as indicated in Table 3, which meets the requirements for commercial thermal insulation coatings. Notably, the lowest thermal conductivity observed in the MGA-10/20/x series foamy coatings is 31 mW/m·K, as determined by the heat flow method (HFM). The thermal conductivity of the foamy coatings decreases as the aerogel content in the system increases, as shown in Figure 5. Compared to typical commercially available coatings, the thermal conductivity of the thermal insulating coatings in this study significantly decreased to below 0.1 W/m·K. Specifically, the average thermal conductivity of MGA-10/20/5, MGA-10/20/7.5, and MGA-10/20/10 was 0.0537, 0.0305, and 0.0277 W/m·K, respectively, at 35 °C. These results suggest that foamy aerogel composites, when precisely designed, have the potential to replace traditional thermal insulation pads.

The temperature difference between the top and bottom surfaces of the coating boards was recorded as indicated in Figure 5’s inset image. The MSA series is more compact compared to the MGA series, resulting in an inferior thermal insulation performance. As a result, further research focused primarily on the MGA series. The corresponding temperature differences for the MGA-10/20/z series were calculated based on the recorded data. Compared to the FCB with a regular MF resin coating without a foamy structure, which showed an average temperature difference of 18.5 °C, the foamy aerogel coating demonstrated an exceptional thermal insulation performance. The MGA-10/20/10 samples exhibited the greatest temperature difference of 54.1 °C after 10 min on the heat plate. The foamy structure provided a 35.6 °C improvement in the temperature difference, acting as an effective thermal barrier coating. Such excellent thermal insulation behavior could find it potential applications in exterior insulation systems for buildings due to its low thermal conductivity, high cohesive strength, fire resistance, and low carbon emission properties. These results confirmed that the foamy aerogel coating is a promising thermal insulation material for the exterior walls of constructions in extreme weather conditions.

These results are consistent with the previous characterization of the microstructure. Since MGA 10/20/10 shows the most uniform pore size distribution, the aerogel-stabilized hierarchical porous structure contributes to the overall reduction in thermal conductivity. The thermal conductivities of MGA 10/20/7.5 and MGA 10/20/10 are similar when the testing temperature exceeds 80 °C, which is due to the limitations of the pore size and the resin component in the composite. When the temperature is low, the thermal conductivity of the aerogel composite is primarily influenced by the insulating behavior of the porous phase. However, at moderate and high temperatures, the resin outweighs the insulating properties of the pores and leads to a greater thermal conductivity at a higher temperature.

## 4. Conclusions

Thus far, we have demonstrated the effectiveness of SiO_2_ aerogel nanoparticles serving as an interface stabilizer in coating composites. The coating composite derived with a uniformly distributed nanoporous structure possesses an extremely low thermal conductivity of 0.036 W/m·K and excellent thermal insulating behavior at 53.4 °C for a block sample of a 1 cm thickness after 10 min of sitting a heating plate. The adhesion, durability, and other essential properties of this coating material must be validated further before its practical application.

This architected foamy aerogel coating provides an applicable measure for enhancing the thermal insulating behavior as well as the fire resistance of exterior installations for buildings with inflammable materials, such as melamine resin. Equally, the foamy resin provides a unique solution in solving the resin’s intrusion into and the collapse of the nanoporous structures of the functional aerogel, which preserves the stunning properties of this nanomaterial. The cost-effectiveness of the raw materials, the easy fabrication procedure, and its remarkable comprehensive performance make this foamy aerogel coating promising for reaching the net-zero emissions goal in the construction and infrastructure sectors.

## Figures and Tables

**Table 1:** Samples of MGA-10/20/x series and MSA-10/20/x.

	Foaming Agent	Melamine Resin Content	4% Foaming Agent Content	SiO_2_ Aerogel Powder Content
MGA-10/20/2.5	GA	10	20	2.5
MGA-10/20/5				5
MGA-10/20/7.5				7.5
MGA-10/20/10				10
MSA-10/20/2.5	SA			2.5
MSA-10/20/5				5
MSA-10/20/7.5				7.5
MSA-10/20/10				10

**Table 2:** Surface area and Barrett–Joyner–Halenda (BJH) desorption pore volume and pore diameter of MGA-10/20/x series and MSA-10/20/x series.

	BET Surface Area (m^2^/g)	BJH Pore Volume (cm^3^/g)	Average Pore Diameter (Å)
MGA-10/20/2.5 ^1^	/	/	/
MGA-10/20/5	297.01 ± 1.31	0.69	57.81
MGA-10/20/7.5	408.95 ± 5.01	0.97	57.07
MGA-10/20/10	417.48 ± 4.47	1.06	58.76
MSA-10/20/2.5	235.93 ± 2.56	0.74	58.56
MSA-10/20/5	239.64 ± 2.53	0.89	66.06
MSA-10/20/7.5	387.42 ± 4.39	1.04	60.25
MSA-10/20/10	442.88 ± 5.13	0.67	59.26

^1^ The MGA-10/20/2.5 sample was too fragile to be tested.

**Table 3:** Thermal conductivity of MGA-10/20/5 and MGA-10/20/7.5 at various temperatures.

Temperature	35	40	60	80	100	200
MGA-10/20/5	0.0537 ± 0.002	0.0540 ± 0.002	0.0569 ± 0.003	0.0595 ± 0.003	0.0644 ± 0.003	0.0649 ± 0.003
MGA-10/20/7.5	0.0305 ± 0.001	0.0317 ± 0.002	0.0351 ± 0.002	0.0379 ± 0.002	0.0385 ± 0.002	0.0410 ± 0.002
MGA-10/20/10	0.0277 ± 0.001	0.0280 ± 0.001	0.0324 ± 0.002	0.0367 ± 0.002	0.0378 ± 0.002	0.0391 ± 0.002

## Data Availability

Data are contained within the article.
